# Evaluation of *Echinacea* Leaf Extract and Allantoin Based Topical Formulations in an Imiquimod-Induced Psoriasis-like Model

**DOI:** 10.3390/medsci14020283

**Published:** 2026-06-01

**Authors:** Cristina Burlou-Nagy (Fati), Annamaria Pallag, Laura Grațiela Vicaș, Octavia Gligor, Tünde Horvath, Beáta Lelesz, Csaba Hegedűs, Imre Leiter, Béla Juhász, Rita Kiss

**Affiliations:** 1 Doctoral School of Biological and Biomedical Sciences, University of Oradea, Universitatii Str. No 1, 410087 Oradea, Romania; burlounagy.cristina@student.uoradea.ro; 2 Department of Pharmacy, Faculty of Medicine and Pharmacy, University of Oradea, P-ta 1 Decembrie No 10, 410073 Oradea, Romania; lvicas@uoradea.ro (L.G.V.); thorvath@uoradea.ro (T.H.); 3 Department of Preclinical Disciplines, Faculty of Medicine and Pharmacy, University of Oradea, P-ta 1 Decembrie No 10, 410073 Oradea, Romania; octavia.gligor@uoradea.ro; 4 Department of Pharmacology and Pharmacotherapy, Faculty of Medicine, University of Debrecen, Nagyerdei Krt 98, 4032 Debrecen, Hungary; lelesz.beata@med.unideb.hu (B.L.); csaba.hegedus@med.unideb.hu (C.H.); leiter.imre@med.unideb.hu (I.L.); juhasz.bela@med.unideb.hu (B.J.); kiss.rita@med.unideb.hu (R.K.)

**Keywords:** topical formulations, *Echinacea* leaf extract, psoriasis, rat model, herbal therapeutics

## Abstract

Background/Objectives: Psoriasis is a chronic inflammatory disorder characterized by epidermal hyperproliferation and immune dysregulation. This study evaluated the topical efficacy of *Echinacea* leaf extract and allantoin compared to a corticosteroid (mometasone furoate) in an imiquimod (IMQ)-induced psoriasis-like model in Sprague-Dawley rats. Methods: Psoriasiform lesions were induced by daily application of 5% IMQ for six days, followed by six weeks of treatment with one of the following formulations: *Echinacea* cream or gel, allantoin cream or gel, and mometasone. Disease severity was assessed using a modified Psoriasis Area and Severity Index (PASI) and histological analysis. Results: All groups developed psoriasis-like lesions; however, *Echinacea* cream produced the most pronounced and sustained reduction in PASI scores, with near-complete macroscopic recovery. *Echinacea* gel and allantoin cream showed moderate effects, while allantoin gel and mometasone exhibited limited efficacy. Histological findings supported these results, with *Echinacea* cream restoring near-normal epidermal architecture. Conclusions: In conclusion, topical *Echinacea* leaf extract formulations, particularly the cream formulation, demonstrated superior efficacy in reducing psoriasis-like skin inflammation and restoring epidermal structure compared to allantoin based treatments and mometasone. These findings suggest that *Echinacea*-derived bioactive compounds may represent promising candidates for the development of alternative or adjunctive therapies for inflammatory skin diseases.

## 1. Introduction

The skin serves as a vital protective barrier against the outer world, making it the biggest organ in the human body. It offers protection from different traumatic incidents and also acts as a regulator for temperature and fluids [1,2]. In the context of experimental and clinical dermatology, skin integrity and its regenerative capacity are key elements in assessing the therapeutic efficacy of topical compounds [3,4].

Natural compounds have been extensively utilized in dermatological treatment and skin maintenance due to their favorable safety profiles and broad spectrum of bioactive properties. Consequently, there has been a growing interest in plant-derived constituents for dermopharmaceutical applications [5,6]. Plant materials can be used externally for skin care objectives, as well as for managing various skin conditions [7,8,9]. Their benefit lies in being gentle yet efficient, safe, and non-toxic, with no adverse reactions.

They can display a variety of characteristics, including medicinal benefits for skin conditions like acne, psoriasis, and eczema, as well as skin care properties such as antioxidants, antibacterial qualities, and moisturizing effects [10].

The regeneration and healing of the skin involve interactions among various cells, including endothelial cells, inflammatory cells, keratinocytes, and fibroblasts [11,12].

In addition, recent studies have further highlighted the increasing interest in natural compounds for dermatological treatments due to their safety profile, proven efficacy, and pharmacological versatility [13,14,15,16].

In a previously published study, we demonstrated that the results of the studies carried out for the determination of total polyphenols content, total flavonoids content, determination of free radical scavenging activity, the HPLC analysis, evaluation of the antimicrobial activity, and evaluation of the wound healing effect of *Echinacea purpurea* (L.) Moench leaf extract using the In Vitro Scratch Assay determined us to choose the leaves of *Echinacea purpurea* (L.) Moench due to the favorable results compared to the other parts of the plant [17]. Their antioxidant, antibacterial, anti-inflammatory, and moisturizing properties confer valuable therapeutic potential in dermato-pharmaceutical formulations [3,18,19]. However, despite these promising findings, there is limited evidence regarding the evaluation of *Echinacea purpurea* leaf extract in in vivo psoriasis models and its incorporation into topical pharmaceutical formulations. This gap highlights the need for further investigation.

Skin diseases are among the most common medical conditions worldwide and represent a substantial global health burden, affecting individuals across all age groups [20,21]. Their development is influenced by a complex interplay between genetic predisposition and environmental factors, including occupational exposure, lifestyle, and pharmacological triggers [22].

Among these conditions, psoriasis is a chronic, immune-mediated inflammatory disease affecting more than 60 million people globally [23]. It is characterized by epidermal hyperproliferation, impaired keratinocyte differentiation, and persistent inflammation, leading to the formation of erythematous, scaly plaques. In addition to its physical manifestations, psoriasis significantly impairs quality of life and is associated with considerable psychosocial burden [23].

Genetic susceptibility plays a key role in psoriasis development, with multiple associated loci identified, particularly PSORS1 [24]. In addition, dysregulation of innate and adaptive immune responses represents a central pathogenic mechanism. At the immunological level, psoriasis is driven by dendritic cell activation and subsequent T-cell-mediated inflammatory responses. Keratinocyte-derived antimicrobial peptides, including LL-37, activate plasmacytoid dendritic cells, leading to interferon production and maturation of myeloid dendritic cells. These, in turn, promote Th1 and Th17 differentiation via cytokines such as TNF-α, IL-12, and IL-23, resulting in a cytokine cascade (including IL-17, IL-21, and IL-22) that induces keratinocyte hyperproliferation, impaired differentiation, and sustained inflammation, ultimately leading to characteristic histopathological changes such as acanthosis, parakeratosis, and inflammatory infiltration [25,26].

Topical corticosteroids remain a cornerstone of psoriasis therapy due to their potent anti-inflammatory effects; however, their long-term use is associated with adverse effects such as skin atrophy, local irritation, and impaired barrier function. These limitations highlight the need for alternative topical treatments that combine anti-inflammatory efficacy with improved safety and tolerability.

In this context, the novelty of the present study lies in the development and evaluation of *Echinacea purpurea* leaf extract-based topical formulations combined with allantoin in an IMQ -induced psoriasis-like rat model.

The aim of this study is to develop and characterize pharmaceutical formulations based on *Echinacea* leaf extracts and to evaluate their therapeutic efficacy in the management of psoriasis. The scope of this study includes formulation development, physicochemical characterization, and in vivo evaluation using an IMQ-induced psoriasis-like rat model.

## 2. Materials and Methods

### 2.1. Materials

The plant material of *Echinacea purpurea* (L.) Moench was obtained from our own cultivated source (Oradea, Romania). Plant specimen was deposited in the Herbarium of the Faculty of Medicine and Pharmacy Oradea and is registered in the NYBG Steere Herbarium under code UOP 05067.

Immediately after harvesting, the leaves (*Echinaceae purpureae folium*, EPF) were air-dried at room temperature and ground using an electric mill equipped with a fast-rotating blade.

The extraction procedure was performed following previously reported methods, with full experimental details provided here in accordance with the ConPhyMP (Consensus statement on the Phytochemical Characterisation of Medicinal Plant extracts) guidelines to ensure reproducibility.

Briefly, 15 g of powdered plant material were subjected to maceration in 150 mL of absolute ethanol (99%, *v*/*v*) for 10 days at room temperature (drug-to-solvent ratio 1:10, *w*/*v*). After maceration, the extract was filtered and the solvent was removed under reduced pressure using a rotary evaporator (R-300, Büchi Labortechnik AG, Flawil, Switzerland) at 35 °C and 300 mbar. The resulting residue was further lyophilized to dryness.

The dried extract was subsequently re-dissolved in 100 mL ethanol and stored at 4 °C in the dark until further use. All steps were performed under standardized conditions to allow reproducibility of the extraction process. The extract was obtained following the same procedure and was subsequently used in the formulation of topical preparations.

The extract was previously characterized in terms of total polyphenol, DPPH assay, flavonoid content, antioxidant activity, and HPLC profile, confirming the presence of key bioactive constituents [17]. The phytochemical characterization of the extract was carried out using a HPLC ACME 3000 Younglin system (SP 930D Young Lin Instrument Co., Ltd., Anyang, Republic of Korea) equipped with a UV730D detector (Young Lin Instrument Co., Ltd., Anyang, Republic of Korea) and a YMC-Pack ODS-AQ column (150 × 4.6 mm, 5 µm particle size). Separation was achieved using an isocratic mobile phase consisting of methanol, water, and acetic acid (30:70:0.3, *v*/*v*/*v*), delivered at a flow rate of 1 mL/min.

Samples were introduced in a volume of 5 µL, and detection was performed at 300 nm. Calibration and detector linearity were assessed using external standards.

As reported in our previous study, the HPLC profile showed that caffeic acid derivatives were found at the highest levels, with chicoric acid (7.09 mg/mL), caftaric acid (4.48 mg/mL), and echinacoside (3.58 mg/mL) predominating, while lower amounts of caffeic acid (1.20 mg/mL) and chlorogenic acid (0.08 mg/mL) were also detected [17].

Prior to analysis, the lyophilized extract was diluted with methanol in a 1:2 ratio (extract:methanol). All experiments were performed using the same extract batch to ensure consistency of the results.

Cetyl alcohol, propylene glycol, glycerin, Synthalen K (carbomer), ethanol, triethanolamine, and sodium lauryl sulfate were used as excipients. The reagents employed in this study were purchased from Elemental (Oradea, Romania), Farmachim 10 SRL (Ploiești, Romania), Sigma-Aldrich (Taufkirchen, Germany), and Fluka (Buchs, Switzerland). All materials complied with the purity specifications indicated in the certificates of analysis provided by the manufacturers.

Synthalen K (Carbopol 940), a synthetic cross-linked poly(acrylic acid) polymer, was selected as the gelling agent for hydrogel preparation due to its ability to produce high viscosity at low concentrations, good skin tolerability, and efficient release of incorporated bioactive compounds through its three-dimensional polymeric network [27,28].

Sodium lauryl sulfate was used as an anionic surfactant and emulsifying agent to stabilize the oil-in-water (O/W) emulsion, facilitating the formation of a homogeneous system and improving the consistency, spreadability, and stability of the formulation.

The cream formulations were prepared as oil-in-water (O/W) emulsions stabilized with sodium lauryl sulfate. This type of emulsion provides good spreadability, ease of removal with water, and suitable tolerability for topical application [29].

### 2.2. Obtaining the Gels

For the preparation of topical semisolid formulations, two distinct bases were selected, a hydrogel and an oil-in-water (O/W) emulsion cream, both chosen for their favorable skin absorption properties and compatibility with bioactive compounds. *Echinacea purpurea* leaf extract and allantoin, known for their anti-inflammatory and skin-regenerating effects, were individually incorporated into each base, resulting in four formulations: hydrogel containing extract, hydrogel containing allantoin, cream containing extract, and cream containing allantoin.

All preparation procedures were performed under controlled conditions to ensure formulation homogeneity, stability, and appropriate consistency. The developed formulations were intended to enable a comparative evaluation of the therapeutic potential of each active compound, depending on the type of vehicle used, within an experimentally induced psoriasis-like model.

### 2.3. Formulation Development

Formulation EG was developed as a hydrophilic gel based on Synthalen K, which was neutralized with triethanolamine to obtain the appropriate gel structure. *Echinacea* leaf extract was used as the active ingredient, while propylene glycol and ethanol were added to enhance solubility and application properties. Distilled water served as the dispersion medium. A 1% *Echinacea* extract gel was obtained (EG).

In a previous study conducted by us, we performed phytochemical characterization of *Echinacea* leaf extract using the HPLC-UV method [17].

Formulation EC was prepared as an emulsion-type cream containing *Echinacea* leaf extract as the active compound. The formulation was based on an emulsifying system consisting of cetyl alcohol and sodium lauryl sulfate, with glycerin acting as a humectant. Distilled water was used as the continuous phase. A 1% *Echinacea* extract cream was obtained (EC).

Formulation AG was prepared as a hydrophilic gel similar in composition to formulation E1, with allantoin incorporated as the active substance at a concentration of 1%. The gel matrix was formed using Synthalen K neutralized with triethanolamine, while propylene glycol and ethanol improved solubility and spreading characteristics. Distilled water completed the formulation. A 1% allantoin gel was obtained (AG).

Formulation AC was prepared as an emulsion-based cream containing allantoin as the active ingredient at a concentration of 1%. The emulsifying system consisted of cetyl alcohol and sodium lauryl sulfate, and glycerin was included to provide moisturizing properties. Sodium lauryl sulfate was incorporated as a surfactant and emulsifying agent to facilitate the formation and stabilization of the oil-in-water cream system and to promote uniform distribution of the formulation components. The concentration was selected to balance emulsification efficiency with acceptable tolerability based on commonly reported usage levels in topical formulations. Distilled water functioned as the dispersion medium. A 1% allantoin cream was obtained (AC).

Full formulation composition and component concentrations are provided in Table 1.

The EG, EC, AG, and AC pharmaceutical formulations were prepared using the following procedures: The hydrogel formulations were prepared by dispersing the polymer (Synthalen K) in a mixture of propylene glycol and distilled water under continuous stirring. After complete polymer hydration, triethanolamine previously dispersed in a mixture of ethanol and water was added to neutralize the system, resulting in gel formation and clarification. For the preparation of oil-in-water (O/W) creams, the oily phase containing cetyl alcohol was heated to 60 °C and dispersed in glycerin. The aqueous phase was prepared by dissolving sodium lauryl sulfate in distilled water and heating it to the same temperature (60 °C). The aqueous phase was gradually added to the oily phase under continuous stirring until a homogeneous emulsion was obtained. After homogenization and cooling to approximately 25 °C, the *Echinacea* leaf extract or allantoin was incorporated into the formulations.

### 2.4. In Vitro Studies on the Transfer of Pharmacologically Active Ingredients from Gels Using a Franz Cell System

Diffusion and membrane permeability studies were carried out using a Franz six-cell diffusion system (system Microette-Hanson, model 57-6AS9, Copley Scientific Ltd., Nottingham, UK). A 0.500 g sample of each formulation was brought into the diffusion cell capsule, being placed on the polysulfon membrane (0.45 μm porous size—Tuffryn^®^, PALL Life Sciences HT-450, lot T72556). The membrane was prepared by hydration in an acceptor solution. The acceptor solution consists of phosphate buffer (pH 7.4) mixed with 30% freshly prepared ethanol. The receiver chamber in each diffusion cell was filled with the prepared solution. The actual diffusion area was 1767 m^2^. The system was maintained at 32 ± 1 °C so that the membrane in the Franz cells was similar to the physiological temperature of the skin. Sampling was carried out at different time intervals (15 min, 30 min, 1, 2, 3, 4, 5, 6, 8, 12, and 24 h). A 0.5 mL sample was taken from the acceptor phase, which was replaced with a fresh receptor medium to maintain a constant volume of acceptor solution in the diffusion cells. The quantity of the pharmacologically active ingredient of the samples (caffeic acid, respectively, allantoin) was determined using the UV-VIS spectral photometric method (PG INSTRUMENTS—model UV-VIS T 92+). In this regard, for the samples with *Echinacea* leaf extract, the protocol involved combining 0.1 mL of samples with 1.7 mL of distilled water and 0.2 mL of freshly diluted Folin–Ciocâlteu reagent (1:10, *v*/*v*), adding 1 mL of 7.5% sodium carbonate solution, after which they were kept at room temperature, protected from light for 2 h. Spectrophotometric determinations were performed at 765 nm for the content of total polyphenols, and for the samples with allantoin, the reading was performed at 227 nm. Each preparation was tested in three specimens, and the data were presented as an average ± SD (standard deviation). The cumulative release rate (CDR) was calculated using Equation (1):

(1) $$CDR\left(\%\right)=\frac{Q_{n}}{Q_{t}}\times 100$$

where Qn was the content of the pharmacologically active ingredient and the total content at time n, and Qt was the initial content of the substance embedded in gels [30,31].

### 2.5. Experimental Animals

Twelve-week-old male Sprague-Dawley rats, weighing 350–400 g, were purchased from Charles River Laboratories International, Inc. (Wilmington, MA, USA) and used as experimental animals. The rats were maintained under standard laboratory conditions at a controlled temperature of 22 ± 2 °C and relative humidity of 50–60%, with a 12 h light/dark cycle. Animals had *ad libitum* access to standard laboratory chow (S8106-S011 SM R/M-Z+H; ssni Spezialdiäten GmbH, Soest, Germany) and drinking water throughout the study. Prior to the initiation of the experimental procedures, all animals underwent a two-week acclimatization period to allow physiological stabilization under the housing conditions.

### 2.6. Experimental Design

**Figure 1:**
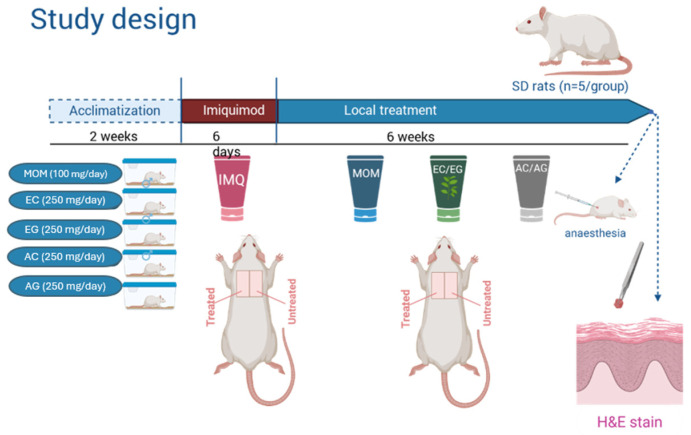
Overview of the Preclinical Study Procedure. The schematic illustration was created with BioRender.com.

Following acclimatization, animals were randomly assigned to five experimental groups (n = 5/group), as shown in Figure 1. To prevent removal of topical formulations and mechanical irritation, animals were housed individually. A 3 × 3 cm dorsal skin area was shaved prior to treatment. Psoriasis-like skin inflammation was induced by daily topical application of 62.5 mg of 5% IMQ cream (Aldara^®^, Viatris Healthcare, Canonsburg, PA, USA, supplied by the institutional pharmacy) to one half of the shaved dorsal skin for six consecutive days, while the contralateral untreated skin served as an internal control within each animal [32,33].

The experimental groups included mometasone furoate (MOM, positive control), *Echinacea* leaf extract cream (EC) and gel (EG), as well as allantoin-based cream (AC) and gel (AG) formulations. Following IMQ induction, topical treatments were administered once daily for six weeks. The MOM group received 100 mg of MOM formulation (Momegen 1 mg/g, Viatris Limited, Canonsburg, PA, USA, supplied by the institutional pharmacy), while EC, EG, AC, and AG groups were treated with 250 mg of the respective formulations, ensuring complete coverage of the treated dorsal skin area (Figure 1). The formulations were stored in closed containers at 4 °C during the experimental period. No preservatives were added to the formulations to avoid potential confounding effects on skin physiology and inflammatory responses. As the preparations were intended for short-term experimental use, freshly prepared batches and controlled storage conditions were applied to minimize microbial contamination. The amount of the mometasone formulation (100 mg/application) was determined based on preliminary pilot experiments and established topical dosing principles for potent corticosteroid preparations, which are typically effective at lower application volumes. This dose was considered pharmacologically relevant within the experimental model, while the higher amounts used for the experimental plant-based formulations were necessary to ensure adequate coverage and delivery of the lower-concentration active compounds.

### 2.7. Macroscopic Evaluation

During the treatment period, daily photographic documentation of the treated skin areas was performed. The severity of skin lesions was assessed using a modified Psoriasis Area and Severity Index (PASI) adapted for experimental models. The PASI scoring system evaluated erythema, scaling, and skin thickness [34]. PASI scoring was performed independently by three investigators based on erythema, scaling, and skin thickening parameters. The obtained scores were averaged and used for subsequent statistical analysis.

### 2.8. Histological Analysis

At the end of the six-week treatment period, the animals were humanely euthanized in accordance with established ethical guidelines. Skin samples were collected from both the treated dorsal area and the contralateral untreated control region. The harvested tissues were fixed and processed according to standard histological procedures. After fixation, the samples were dehydrated, embedded in paraffin, sectioned, and stained with hematoxylin–eosin (H & E) to allow detailed morphological evaluation.

Histological evaluation, semi-quantitative scoring, and morphometric image analysis were independently performed by two investigators according to predefined morphological criteria in order to minimize observer-related bias and improve reproducibility.

Histological analysis focused on the quantitative assessment of epidermal thickness and stratum corneum thickness. In addition, the ratio of stratum corneum thickness to total epidermal thickness was calculated as an indicator of structural alterations associated with psoriasis-like skin pathology. Morphological evaluation was performed using light microscopy (Nikon Eclipse 80i and Nikon DS-Fi3 microscope camera-Nikon Corporation, Tokyo, Japan) combined with digital image analysis (NIS-Elements Viewer Imaging Software version 5.21-Nikon Corporation, Tokyo, Japan) to ensure objective and reproducible evaluation of the histological parameters.

Histological alterations were further assessed using a semi-quantitative scoring system evaluating epidermal thickness (acanthosis), hyperkeratosis/parakeratosis, rete ridge elongation (epidermal architectural distortion), and dermal inflammatory infiltration. Each parameter was scored on a scale from 0 to 3 (0 = absent/normal, 3 = marked), and a composite score (range: 0–12) was calculated as the sum of all parameters. Scoring was performed using the contralateral untreated skin within the same animal as an internal morphological reference. However, the contralateral untreated skin areas were not included in the statistical analysis as repeated within-animal measurements. The presence of Munro micro-abscesses was assessed qualitatively; none were observed in any of the samples.

### 2.9. Statistical Analysis

Statistical analyses were performed using GraphPad Prism 9.1.2. software. Prior to analysis, data distribution was assessed using the Shapiro–Wilk and Kolmogorov–Smirnov tests to evaluate normality. Longitudinal PASI data were analyzed using two-way repeated-measures ANOVA, with time and treatment group as factors, including assessment of interaction effects.

Histological scoring data were analyzed using the Kruskal–Wallis test followed by Dunn’s multiple comparisons test, as the scoring system represented semi-quantitative ordinal data. Quantitative morphometric data were analyzed according to their variance characteristics. Homogeneity of variances was assessed using the Brown–Forsythe test. In the case of unequal variances, as observed for epidermal thickness, Welch’s ANOVA was applied, followed by Games–Howell post hoc comparisons. For parameters with homogeneous variances, including stratum corneum thickness and the stratum corneum to epidermis ratio, standard one-way ANOVA was used. Data are presented as mean ± SD. The value of *p* < 0.05 was considered statistically significant.

### 2.10. Ethical Approval

All experimental procedures involving animals were conducted in accordance with the relevant national and institutional guidelines for the care and use of laboratory animals. The study protocol was reviewed and approved by the University of Debrecen Workplace Animal Welfare Committee (Debreceni Egyetem Munkahelyi Állatjóléti Bizottsága) under approval number 13-1/2025/DEMÁB.

## 3. Results

### 3.1. Topical Formulations

All four topical formulations—hydrogels and creams containing either *Echinacea purpurea* leaf extract or allantoin—were successfully prepared under controlled laboratory conditions, resulting in homogeneous and stable products with consistent macroscopic characteristics.

The carbopol-based hydrogels containing *Echinacea purpurea* leaf extract (EG) and allantoin (AG) displayed a clear and uniform appearance, along with high viscosity even at low polymer concentrations, confirming the expected gelling performance of Synthalen K and the efficiency of triethanolamine neutralization.

**Figure 2:**
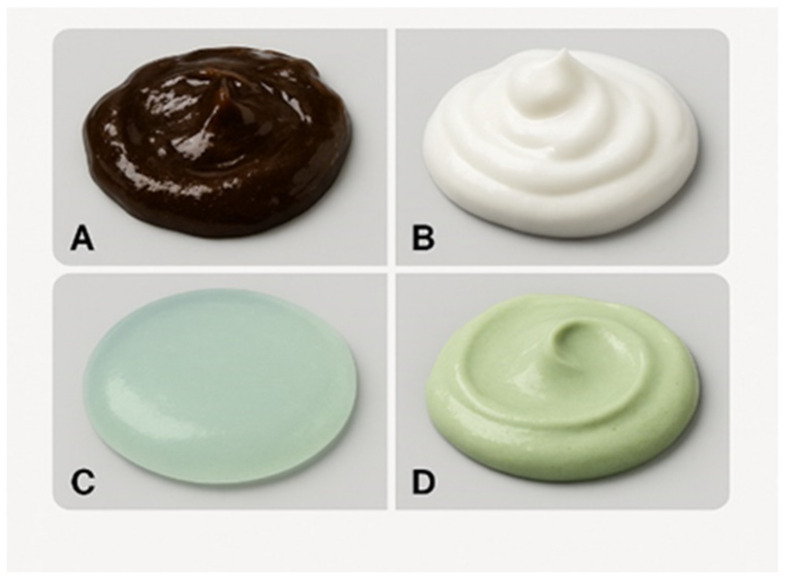
Macroscopic appearance of topical formulations. (**A**)—Hydrogel containing *Echinacea purpurea* leaf extract (EG). (**B**)—Cream containing allantoin (AC). (**C**)—Hydrogel containing allantoin (AG). (**D**)—Cream containing *Echinacea purpurea* leaf extract (EC).

The oil-in-water emulsion creams containing *Echinacea purpurea* leaf extract (EC) and allantoin (AC) exhibited a smooth, easily spreadable texture and remained stable after incorporation of the active compounds (Figure 2). This indicates that the sodium lauryl sulfate-based emulsifying system ensured adequate phase dispersion and structural integrity.

No signs of phase separation, precipitation, or incompatibility were observed in any of the formulations, demonstrating good physicochemical compatibility between the active ingredients and the selected bases.

The addition of *Echinacea* extract and allantoin did not compromise the structural properties of either the hydrogel or the cream matrices, confirming that both systems are suitable carriers for these bioactive compounds.

Differences in viscosity, transparency, and spreadability between hydrogels and creams were consistent with the known behavior of carbopol networks versus oil-in-water emulsions, supporting their use in subsequent comparative evaluation within the psoriasis-like model.

### 3.2. Results of the In Vitro Transfer of Pharmacologically Active Ingredients from Gels Using a Franz Cell System

The release of IAF from gels was evaluated using Franz diffusion cells. Caffeic acid was chosen as a marker for the evaluation of gel release due to its representativeness for the phenolic compounds in *Echinacea* extract, chemical stability, and ease of analytical determination. In addition, it exhibits physicochemical properties suitable for diffusion studies and a significant contribution to the biological activity of the extract. The quantities of caffeic acid released over time are shown in Table 2, and the allantoin keys are in Table 3. The calibration curves for each substance were used to determine the respective caffeic acid and allantoin released from the gels. The calibration curve for caffeic acid was used to interpret the results y = 0.0644x + 0.0365, R2 = 0.9997, where y—solution absorption (u.a) at 325 nm, and x—concentration in caffeic acid (mmol/L). In allantoin, the calibration curve was used to interpret the results y = 0.0718x + 0.0256, R2 = 0.9991, where y—solution absorption (u.a) at 227 nm, and x—concentration in allantoin (mmol/L). From the analysis of the graphs obtained, it can be observed that the release of the embedded caffeic acid occurs gradually, with the greatest amount taking place in the first 7 h, after which the trajectory of the curves becomes approximately parallel to the abscess since the caffeic acid release is greatly reduced. With regard to allantoin, it was found that most of the embedded substance was released within the first 8 h.

The release profile followed the Higuchi model, indicating a predominant diffusion mechanism Table 4.

**Figure 3:**
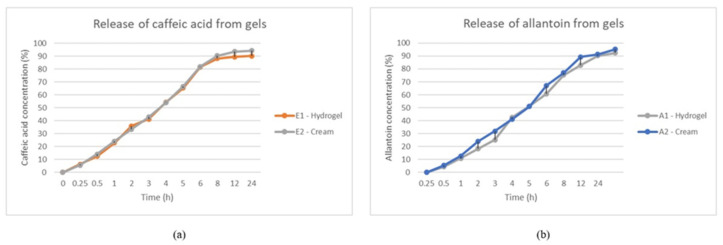
In vitro release profiles of pharmacologically active compounds from gel formulations. (**a**) Gel formulation containing *Echinacea* leaf extract; (**b**) gel formulation containing allantoin. Data are presented as mean ± SD. Statistical analysis was performed using one-way ANOVA followed by Tukey’s multiple comparisons test. Statistical significance was accepted at *p* < 0.05.

In Figure 3, the amount of the active ingredient released was expressed as a percentage and graphically represented in terms of time (h). Based on the in vitro ceding results, the diffusion of *Echinacea* leaf extract from gels through the membrane took place in the first 8 h. We were able to detect over 90% caffeic acid in the cream containing the sodium lauryl sulfate emulsifier compared to 88% caffeic acid in the carbopol-based hydrogel. In the case of allantoin-containing gels, we detected 89% allantoin in the cream with anionic emulsifier compared to what was detected in the hydrogel, namely 82% allantoin at 8 h. The graphical representations of the caffeic acid and allantoin release from the gels are illustrated in Figure 3a,b. We note that in vitro release testing provides a simplified representation of formulation performance and does not fully capture skin interactions or in vivo administration dynamics.

In the case of the gels containing *Echinacea* leaf extract, we observed a gradual and uniform shedding of the two products. We mention that from the cream containing the emulsifier, the highest amount of caffeic acid yield occurs at 8 h, 90%. Allantoin in gels has a similar yield to caffeic acid. We see from Figure 3 that the yield in the cream is a large amount at 8 h, being 91%. We believe that the pharmacologically active ingredients are gradually released from each base of the ointment, but the cream provides a greater release of hydrogels for both *Echinacea* leaf extract and allantoin.

### 3.3. Macroscopic Evaluation of Psoriasis-like Lesions

Topical application of IMQ successfully induced psoriasis-like skin inflammation in all experimental animals. After six days of IMQ treatment, the affected skin areas exhibited characteristic erythema, scaling, and thickening, consistent with previously described IMQ-induced psoriasis models. This was reflected by a rapid increase in PASI scores during the early phase of the experiment, with values rising between weeks 1 and 3 and reaching peak severity around weeks 3–4.

Following this peak, divergent trends were observed among treatment groups. In EC and EG groups, PASI scores showed a consistent and progressive decrease from week 4 onward, indicating marked improvement in macroscopic skin condition. By the end of the treatment period, both formulations demonstrated a substantial reduction in lesion severity, approaching near-baseline values.

AC and AG also exhibited a decrease in PASI scores after the peak phase; however, the magnitude of improvement was more moderate and variable compared to EC and EG. Although partial regression of erythema and scaling was observed, residual inflammatory features persisted at later time points.

In contrast, MOM displayed a distinct pattern. After the initial increase, PASI scores remained elevated and showed a secondary increase at later time points, particularly around week 6, indicating a lack of sustained therapeutic effect. In some animals, inflammatory changes extended beyond the IMQ-treated area.

**Figure 4:**
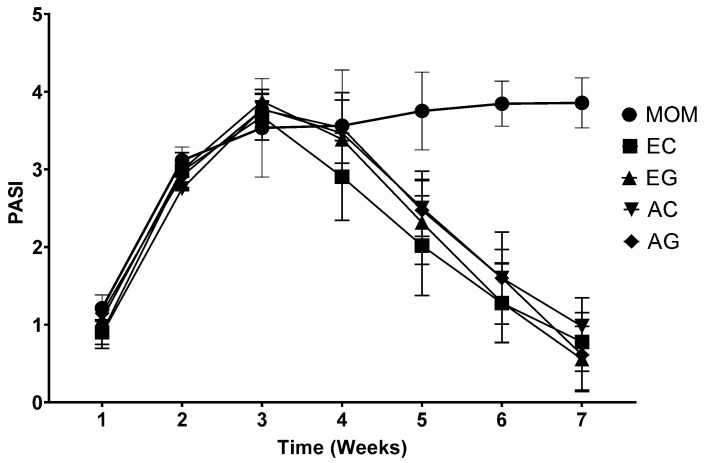
Time-dependent changes in PASI scores during the treatment period. IMQ treatment induced progressive increases in PASI scores until weeks 3–4, followed by divergent treatment responses among groups. EC and EG demonstrated the most pronounced reduction in lesion severity during the late treatment phase, whereas MOM remained persistently elevated. Data are presented as mean ± SD (n = 5 animals/group). Statistical analysis was performed using two-way repeated measures ANOVA followed by Tukey’s multiple comparisons test.

These findings indicate that EC and EG produced the most pronounced macroscopic improvement, whereas AC and AG showed intermediate efficacy, and MOM resulted in limited and inconsistent therapeutic response (Figure 4).

Macroscopic severity of psoriasis-like skin lesions was evaluated using a modified PASI scoring system over a 7-week period. All groups exhibited a rapid increase in PASI scores during the induction phase, reaching peak values around week 3–4. Subsequent reduction in PASI scores was observed in the *Echinacea*-treated groups (EC, EG), indicating progressive improvement. Allantoin-treated groups (AC, AG) showed moderate decreases in lesion severity. In contrast, the MOM-treated group maintained elevated PASI values with no sustained improvement. Data are presented as mean ± SEM.

### 3.4. Histological Findings

Histological analysis was performed using paired comparison between contralateral untreated skin and IMQ-exposed treated skin within each experimental group. The contralateral skin consistently exhibited normal epidermal architecture, whereas IMQ-treated regions showed characteristic psoriasiform alterations, including epidermal hyperplasia, hyperkeratosis, and dermal inflammatory infiltration.

**Figure 5:**
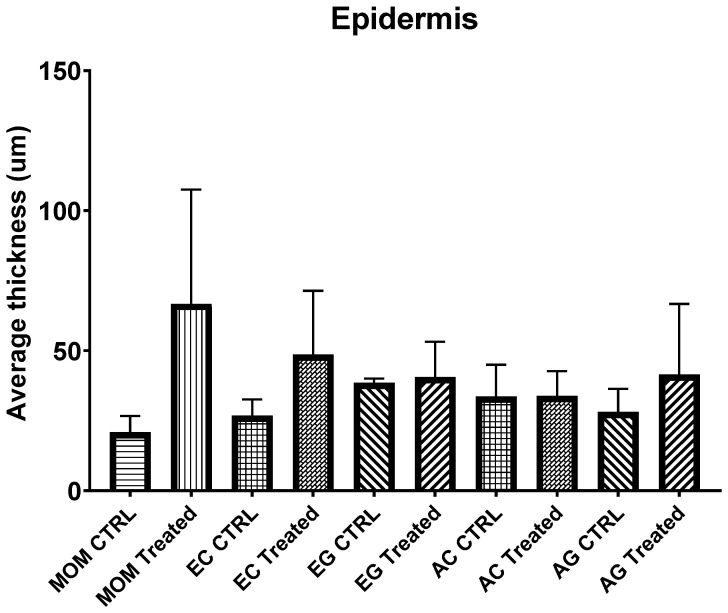
Epidermal thickness. Epidermal thickness in contralateral control and IMQ-treated skin across experimental groups. Welch’s ANOVA revealed a significant overall difference among groups (W (9, 12.89) = 4.586, *p* = 0.0070). However, Games–Howell post hoc analysis did not identify statistically significant pairwise differences. Data are presented as mean ± SD (n = 4–5 animals/group).

Quantitative assessment of epidermal thickness revealed a significant overall difference among groups when analyzed using Welch’s ANOVA (W (9,12.89) = 4.586, *p* = 0.0070), accounting for unequal variances. However, post hoc analysis using the Games–Howell test did not identify statistically significant pairwise differences. These findings suggest that, despite detectable global variation, substantial within-group variability and limited sample size may reduce the statistical power to resolve individual group differences. Descriptive analysis indicated a tendency toward increased epidermal thickness in the mometasone-treated group, whereas the *Echinacea*-based and allantoin-treated groups exhibited lower or intermediate values (Figure 5). In contrast, analysis of stratum corneum thickness did not reveal significant differences among groups (one-way ANOVA: F (9,33) = 0.9442, *p* = 0.5015), and variance homogeneity was confirmed (Brown–Forsythe test: *p* = 0.3428). These results indicate that IMQ-induced alterations and subsequent treatments did not significantly affect the thickness of the cornified layer (Figure 5).

**Figure 6:**
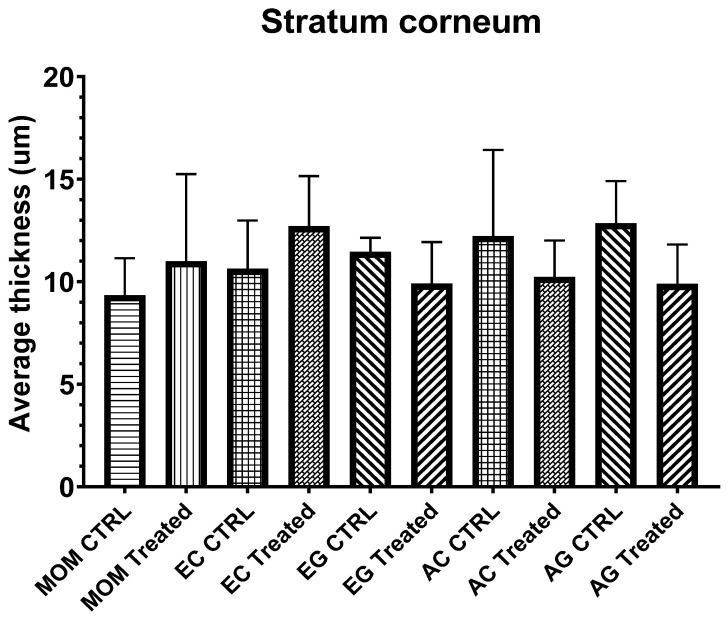
Stratum corneum thickness. Stratum corneum thickness across experimental groups. No statistically significant differences were observed among groups (one-way ANOVA: F (9,33) = 0.9442, *p* = 0.5015). Variance homogeneity was confirmed by the Brown–Forsythe test. Data are presented as mean ± SD (n = 4–5 animals/group).

**Figure 7:**
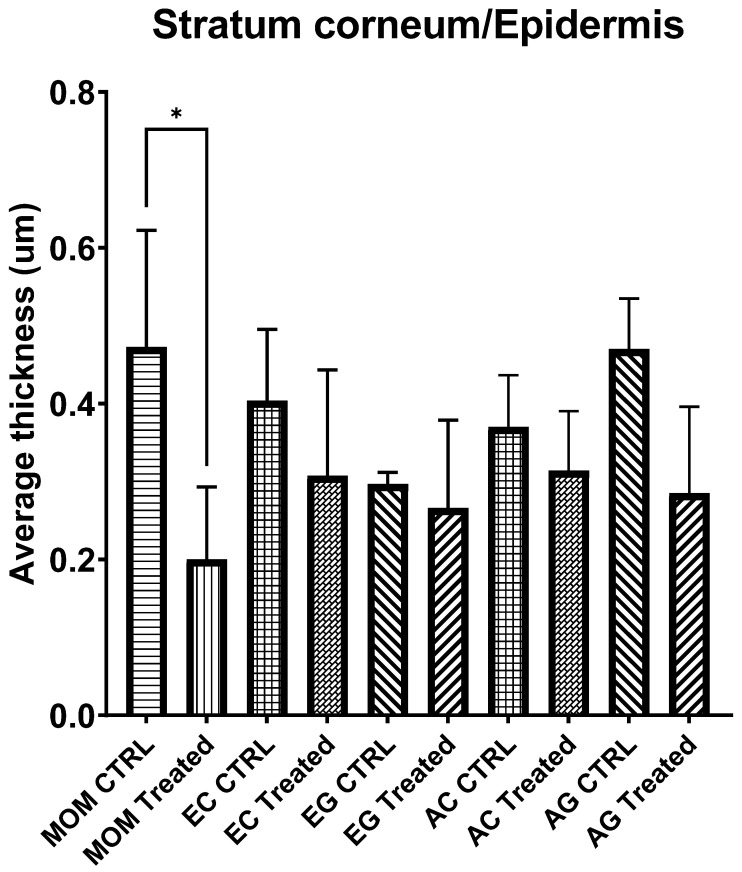
Stratum corneum to epidermis thickness ratio. Ratio of stratum corneum to epidermal thickness across experimental groups. A significant difference was observed (one-way ANOVA: F (9,33) = 3.321, *p* = 0.0054), with homogeneous variances. Lower ratio values in the mometasone-treated group indicate disproportionate epidermal thickening, whereas higher ratios in *Echinacea*-based and allantoin-treated groups reflect improved epidermal organization. Data are presented as mean ± SD (n = 4–5 animals/group). * *p* < 0.05 versus MOM CTRL (Tukey’s multiple comparisons test).

Furthermore, evaluation of the stratum corneum-to-epidermis thickness ratio demonstrated a significant difference among experimental groups (one-way ANOVA: F (9,33) = 3.321, *p* = 0.0054), with confirmed homogeneity of variances (Brown–Forsythe test: *p* = 0.6748) (Figure 6). This parameter revealed clear treatment-related differences, with the mometasone-treated group showing reduced ratio values, indicative of disproportionate epidermal thickening. In contrast, *Echinacea*-based and allantoin-treated groups exhibited higher ratio values, suggesting a more balanced epidermal structure and improved normalization of epidermal differentiation (Figure 7).

Taken together, these findings indicate that while absolute stratum corneum thickness remained unchanged and epidermal thickness showed only global differences, the stratum corneum-to-epidermis ratio proved to be the most sensitive histological parameter for detecting treatment-related effects.

### 3.5. Group-Level Histological Outcomes

**Figure 8:**
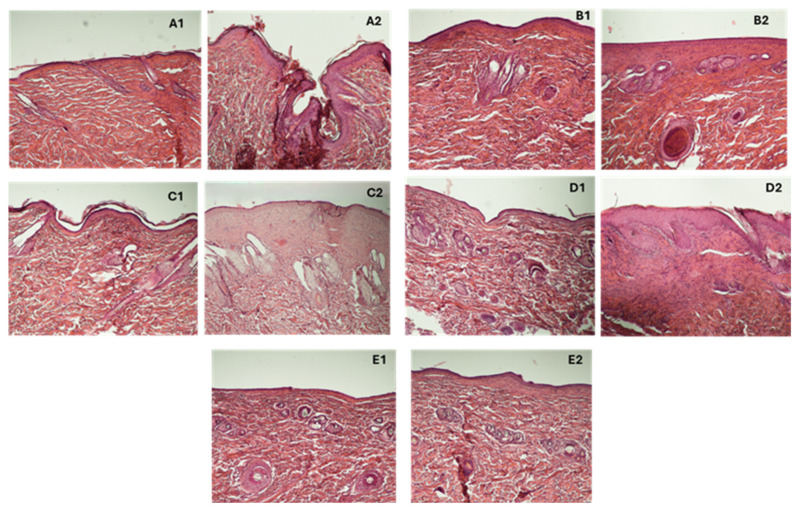
Histological evaluation of treatment effects in the IMQ-induced psoriasis-like model, where (**A1**,**A2**)—MOM group; (**B1**,**B2**)—EC group; (**C1**,**C2**)—EG group; (**D1**,**D2**)—AC group; (**E1**,**E2**)—AG group.

Group-level histological evaluation was performed using a semi-quantitative scoring system incorporating key psoriasiform features, including epidermal hyperplasia, hyperkeratosis, inflammatory infiltration, and rete ridge elongation. Representative hematoxylin–eosin-stained sections are shown alongside quantitative scoring data (Figure 8).

Representative hematoxylin–eosin-stained skin sections are shown for each treatment group. For every group, contralateral untreated (1) and treated (2) skin areas are presented (A–E). Groups are displayed in the order of treatment allocation (MOM, EC, EG, AC, AG), consistent across all analyses. The MOM group (A1–A2) showed partial normalization of epidermal architecture with residual acanthosis and rete ridge elongation. The EC group (B1–B2) demonstrated near-complete restoration of normal skin morphology. The EG group (C1–C2) exhibited moderate improvement with persistent epidermal thickening and inflammatory features. The AC group (D1–D2) showed partial regression with residual psoriasiform alterations. The AG group (E1–E2) retained pronounced epidermal hyperplasia, hyperkeratosis, and inflammatory infiltration, indicating minimal therapeutic effects. Scale bar: 50 µm.

Consistent with the morphological observations, histological scoring revealed differences in treatment response among groups. The AG group exhibited the highest scores, reflecting pronounced epidermal hyperplasia, elongated rete ridges, and persistent inflammatory infiltration, indicating minimal therapeutic effects. In contrast, the EC group demonstrated the lowest scores, corresponding to near-complete normalization of epidermal architecture with minimal residual pathological features.

Intermediate responses were observed in the EG, AC, and MOM groups, which showed partial attenuation of psoriasiform alterations. These samples were characterized by moderate epidermal thickening and reduced, yet still detectable, inflammatory infiltration. The MOM group, in particular, displayed variable responses with persistent structural abnormalities, including acanthosis and rete ridge elongation.

**Figure 9:**
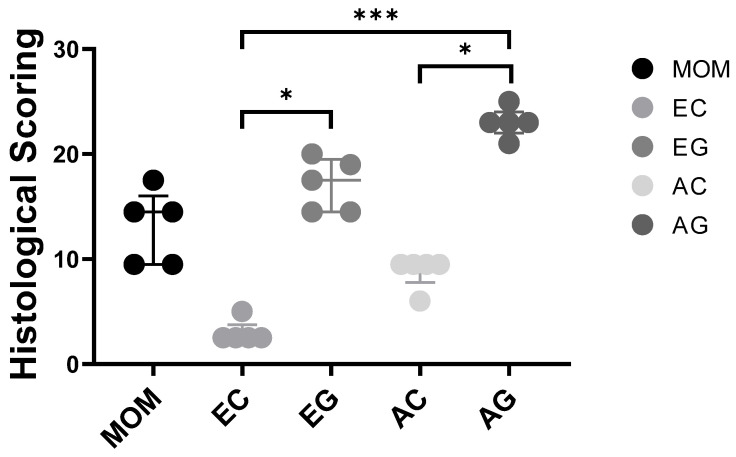
Histological scoring of psoriasiform alterations across experimental groups. Scoring was performed based on key morphological features, including epidermal hyperplasia, hyperkeratosis, inflammatory cell infiltration, and rete ridge elongation. Individual values are shown for each animal together with group distributions (n = 5 animals/group). Normality of each dataset was evaluated using Shapiro–Wilk and Kolmogorov–Smirnov tests prior to statistical analysis. Due to the semi-quantitative ordinal nature of the scoring system, group differences were analyzed using the Kruskal–Wallis test followed by Dunn’s multiple comparisons test. The AG group exhibited the highest scores, indicating severe and persistent psoriasiform pathology. In contrast, the EC group showed minimal scores, consistent with near-complete normalization of skin structure. The MOM, EG, and AC groups displayed intermediate scores, reflecting partial therapeutic effects with residual histological alterations. Statistical significance between groups is indicated as * *p* < 0.05, *** *p* < 0.001.

Non-parametric analysis using the Kruskal–Wallis test followed by Dunn’s multiple comparisons test demonstrated significant differences between EC and EG, EC and AG, as well as AC and AG groups (Figure 9). Collectively, these findings indicate marked histological improvement in the EC group, whereas the AG group displayed the weakest therapeutic response.

## 4. Discussion

Cream and hydrogel formulations were selected because of their widespread use in dermatological therapy and their distinct physicochemical properties, which substantially influence skin hydration, penetration, retention, and local bioavailability of active compounds. The choice of cream and hydrogel formulations was guided by their widespread use in dermatological practice and their distinct physicochemical properties, which influence skin absorption and therapeutic efficacy [35]. Creams, due to their semi-occlusive nature and lipid-rich composition, enhance skin hydration and prolong contact time with the active ingredient, making them suitable for dry or inflamed skin [36]. Hydrogels, on the other hand, offer a non-greasy alternative with excellent cooling and soothing effects, often preferred in acute inflammatory conditions [37]. By comparing these two vehicles, we aimed to evaluate not only the biological activity of *Echinacea purpurea* extract, but also the impact of formulation type on treatment outcomes. The exclusion of other forms, such as ointments or lotions, was intentional, as they either lack patient compliance or do not provide optimal delivery characteristics for the targeted skin condition.

The present study evaluated the therapeutic efficacy of topical *Echinacea* leaf extract-containing formulations and alantoin-based preparations in an IMQ-induced psoriasis-like rat model. This model is widely recognized for its ability to reproduce key immunopathological features of human psoriasis through activation of the TLR7/IL-23/IL-17 axis, resulting in epidermal hyperplasia, hyperkeratosis, and inflammatory cell infiltration [38,39,40,41]. Consistent with previous reports, IMQ treatment in the present study induced characteristic psoriasiform changes, including erythema, scaling, and marked epidermal thickening.

The therapeutic responses differed substantially among treatment groups. Both macroscopic (PASI) and histological assessments indicated pronounced therapeutic effects in the EC-treated group, accompanied by near-complete normalization of epidermal architecture and minimal residual inflammatory changes. This group demonstrated a pronounced and sustained reduction in lesion severity, accompanied by near-complete normalization of epidermal architecture and minimal residual inflammatory changes. In contrast, the EG formulation showed a moderate but less consistent improvement, suggesting that formulation-dependent factors such as skin penetration and local retention may critically influence therapeutic efficacy despite identical active constituents. This observation is in line with previous studies demonstrating that topical delivery systems significantly affect the bioavailability and pharmacodynamic performance of active compounds in inflammatory skin conditions [42,43]. The observed differences between cream and gel formulations may be explained by their distinct physicochemical properties. The use of sodium lauryl sulfate (SLS) was intentional, as the formulations were designed using conventional and widely employed pharmaceutical bases to provide a standardized and reproducible framework for evaluating the incorporated compounds. However, SLS and other excipients may influence skin barrier properties and local irritation, which could contribute to the observed responses in the psoriasis-like model. Therefore, the potential impact of formulation components should be considered when interpreting the results, particularly in the absence of vehicle-only controls. We selected SLS as an anionic surfactant as an emulsifier in semisolid formulations because it reduces the interfacial tension between the oily and aqueous phases, as a very good stabilizer of the emulgel, improves the distribution and homogeneity, and enhances the homogeneous dispersion of bioactive compounds. Another aspect that we considered important is that it determines the increase in penetrability through the stratum corneum of the skin. This is particularly relevant in psoriasis, since many antipsoriatic agents (corticosteroids, vitamin D analogues, retinoids, phytocompounds) are poorly soluble in water and require efficient delivery systems. We note that low concentrations of surfactants in topical formulations for psoriasis determine the improvement of the bioavailability of active substances, penetration into hyperkeratotic psoriatic plaques, and better adherence to treatment of patients. SLS can transiently modify the barrier function of the stratum corneum by interacting with keratin and epidermal lipids, thus increasing permeability. This may improve the topical delivery of phytocompounds with anti-inflammatory properties. Thus, the mechanism is advantageous from a pharmacotechnical point of view in hyperkeratotic lesions, where penetration of substances is often poor.

The Carbopol-based gel contained ethanol and propylene glycol, which can act as penetration enhancers but may also disrupt the skin barrier and alter local tissue responses. In contrast, the oil-in-water cream formulation, containing glycerin and an emulsified lipid phase, likely provided improved hydration and retention of active compounds within the skin. These differences may contribute to the superior therapeutic efficacy observed with the cream formulation.

Allantoin-based treatments exhibited intermediate effects. Both AC and AG formulations resulted in partial attenuation of psoriasiform alterations; however, residual epidermal hyperplasia, rete ridge elongation, and inflammatory infiltration remained evident. Notably, the AG group consistently displayed the highest histological scores, indicating the weakest therapeutic response among all treatments. This finding is consistent with the established pharmacological profile of allantoin, which primarily supports epidermal regeneration and barrier restoration rather than exerting strong anti-inflammatory effects [44,45]. Consequently, while structural improvement was observed, inflammatory processes were not fully suppressed.

Unexpectedly, the mometasone-treated group did not demonstrate consistently greater efficacy than the experimental formulations. Although moderate reductions in PASI scores and partial histological improvement were observed, these effects were not consistently greater than those seen in the EC group, and structural abnormalities persisted. Moreover, PASI dynamics indicated a lack of sustained improvement over time. This discrepancy from clinical expectations may be attributed to differences in treatment duration, dosing regimen, or species-specific pharmacodynamic responses. Similar variability in corticosteroid responsiveness has been reported in experimental psoriasis models, where prolonged exposure may alter local immune regulation or lead to reduced therapeutic responsiveness [46]. Although a lower amount of formulation was applied in the mometasone-treated group, the selected dose was determined based on preliminary pilot experiments and standard dosing considerations for potent topical corticosteroids. This amount provided consistent coverage of the treated skin area and was considered pharmacologically relevant within the conditions of the present model, although differences in formulation composition, application volume, and local exposure cannot be completely excluded. Furthermore, interpretation of comparative treatment efficacy should take into account differences in formulation amount and vehicle composition, as the experimental preparations required larger application volumes to achieve consistent coverage of the treated skin area under the conditions of the present model.

The histological scoring results were in agreement with the morphometric analysis, although not all parameters exhibited equal sensitivity. Although histological scoring represented semi-quantitative ordinal data, non-parametric analysis confirmed the overall pattern of treatment-related differences observed across the experimental groups. Stratum corneum thickness did not show significant intergroup differences, while epidermal thickness demonstrated considerable variability despite observable differences between groups and therefore required variance-corrected statistical approaches. Under the conditions of the present study, the stratum corneum to epidermis ratio appeared to represent a comparatively sensitive indicator of structural normalization. However, these findings should be interpreted cautiously, as not all morphometric parameters consistently supported clear hierarchical separation between treatment groups. Significant alterations in this parameter were primarily observed in the mometasone-treated group, suggesting persistent imbalance in epidermal differentiation rather than uniform treatment failure across all groups.

The pronounced efficacy of the *Echinacea* cream formulation may associate with both the biological activity of its phytochemical components and formulation-related factors, potentially influencing local exposure and retention. *Echinacea*-derived bioactive compounds, including polyphenols and alkylamides, have been shown to modulate key inflammatory signaling pathways such as NF-κB and suppress the production of pro-inflammatory cytokines, including TNF-α, IL-17, and IL-23 [47,48,49]. These mechanisms are highly relevant in psoriasis, where cytokine-driven inflammation promotes keratinocyte hyperproliferation, impaired differentiation, and inflammatory cell infiltration through activation of the IL-23/IL-17 axis. In addition, the antioxidant properties of polyphenolic compounds may attenuate oxidative stress, which is known to contribute to the maintenance and amplification of chronic psoriatic inflammation [50]. The observed normalization of epidermal architecture may also indicate partial restoration of keratinocyte differentiation and barrier homeostasis. Collectively, these effects may explain the marked histological normalization and sustained macroscopic improvement observed in the EC-treated group. However, it is important to emphasize that, in the absence of direct measurements of inflammatory mediators, the present results should be interpreted as phenotypic and histological outcomes rather than direct evidence of specific immunomodulatory mechanisms.

In line with recent studies, plant-derived bioactive compounds have been shown to attenuate epidermal hyperproliferation and inflammatory responses in IMQ-induced models [47,48,49]. Furthermore, the superior performance of the cream formulation compared to the gel observed in the present study underscores the importance of formulation characteristics. While in vitro release profiles provide some indication of formulation behavior, they do not fully capture the complexity of skin delivery and local pharmacokinetics. It is well established that vehicle composition significantly influences skin penetration, retention, and local bioavailability of active compounds, thereby affecting therapeutic outcomes [50,51].

From a translational perspective, these findings suggest that *Echinacea*-based topical formulations may represent a promising alternative or adjunctive strategy for the management of psoriasis-like inflammatory skin conditions. However, further studies are required to elucidate the underlying molecular mechanisms, optimize formulation-dependent bioavailability, and evaluate long-term efficacy and safety in clinically relevant settings.

Although the study provides a comprehensive assessment of treatment performance, several methodological considerations should be acknowledged. The formulations employed were intentionally simple, which may influence the extent of skin penetration and local retention of active compounds. A limitation of this study is that both formulations contained multiple excipients, including ethanol, propylene glycol, glycerin, surfactants, and emulsifiers, which may independently affect skin barrier function, hydration, permeability, and local irritation. These components are not biologically inert and may therefore contribute to the observed effects alongside the incorporated active compounds. However, because similar excipient classes were present across the investigated formulations, their influence is unlikely to fully account for the observed differences between cream and gel formulations. Nevertheless, in the absence of vehicle-only controls, excipient-related effects cannot be completely separated from those of the active ingredients. For instance, the Carbopol-based gel containing ethanol and propylene glycol may subtly modulate barrier properties, whereas the cream, enriched with lipids and humectants, may offer improved compatibility with the skin barrier. Consequently, part of the observed variability in therapeutic outcomes may reflect formulation-related characteristics in addition to the intrinsic activity of the compounds. Furthermore, differences in applied formulation amounts and physicochemical vehicle properties may have influenced topical exposure, penetration, and local bioavailability. Although the mometasone dose was selected based on preliminary pilot experiments and standard corticosteroid dosing considerations, direct quantitative comparison between the corticosteroid and experimental formulations should be interpreted cautiously.

Therapeutic improvements emerged progressively, a pattern likely shaped by both pharmacodynamic properties and formulation-dependent bioavailability. In a clinical context, such a gradual onset may have implications for long-term adherence. The preclinical IMQ-induced psoriasis-like model captures key features of human disease, yet species-specific differences remain possible [52]. Preventive effects were not explored, and mechanistic insights at the molecular level were beyond the scope of this study and should be further investigated in future research of this work, meaning that some interpretations rely on previously published evidence. The modest sample size may also limit the detection of subtle intergroup differences.

Despite these considerations, the study benefits from a rigorous design and the integration of macroscopic, histological, and morphometric analyses, offering a robust and nuanced evaluation of treatment efficacy and highlighting meaningful formulation-dependent effects.

## 5. Conclusions

In conclusion, this study provides in vivo evidence that topical *Echinacea* formulations can reduce psoriasis-like manifestations and associate histopathological changes in the imiquimod-induced model, with the cream formulation demonstrating the most pronounced pharmacological effects among the formulations tested. The comparative differences observed between the cream and hydrogel formulations suggest that the vehicle composition may substantially influence the therapeutic performance by modulating local retention, skin hydration, and cutaneous availability of bioactive compounds. Although sodium lauryl sulfate (SLS) is recognized by the European Medicines Agency (EMA) as a potential irritant capable of increasing skin dryness under certain conditions, the inclusion of SLS at 1% as a stabilizing agent in the hydrogel formulation did not produce any visible adverse effects in the present study. On the contrary, the pharmacological and histological results obtained support the working hypothesis that this concentration could have contributed to an increased epidermal permeability and improved local availability of phytoconstituents without compromising skin integrity in the experimental setting. However, the absence of control groups with exclusive use limits the ability to fully differentiate the contribution of the active compounds from the intrinsic effects of the pharmaceutical bases and excipients. Therefore, the observed therapeutic responses should be interpreted as formulation-dependent results, rather than as effects mediated exclusively by the extract. Overall, these findings highlight the critical role of formulation design and excipient selection in optimizing the efficacy of topical herbal therapies for inflammatory skin conditions such as psoriasis.

## Figures and Tables

**Table 1:** The composition of the formulations is expressed as percentages (%).

The Composition of the Samples	Sample			
	EG (%)	EC (%)	AG (%)	AC (%)
*Echinacea* Leaf Extract	1	1	-	-
Allantoin	-	-	1	1
Synthalen K	1		1	-
Propilenglycol	20	-	20	-
Glycerin	-	15	-	15
Ethyl alcohol 96% (*w*/*v*)	10	-	10	-
Triethanolamine	1	-	1	-
Cetyl alcohol	-	12		12
Sodium lauryl sulfate	-	1		1
Distilled water	67	71	67	71

**Table 2:** The percentage of caffeic acid and allantoin released from the hydrogels and creams with *Echinacea* Leaf Extract and allantoin.

Percentage of CAFFEIC ACID Released (%)	Time (h)										
	0.5	1	2	3	4	5	6	7	8	12	24
EG	6.231	12.543	22.932	35.871	41.286	54.365	65.434	81.604	88.152	89.465	90.173
EC	5.650	14.160	24.171	33.353	42.796	53.892	66.615	82.016	90.295	93.602	94.271

**Table 3:** The percentage of AL released from the 2 allantoin gels.

Percentage of ALLANTOIN Released (%)	Time (h)										
	0.5	1	2	3	4	5	6	7	8	12	24
AG	4.287	10.878	18.145	25.024	42.682	51.223	60.742	75.178	82.972	90.240	92.316
AC	5.286	12.614	23.874	31.961	41.213	50.911	67.290	77.014	89.415	91.241	95.276

**Table 4:** Kinetic parameters of release.

Formulas	Parameter	Value	R2
**EG**	kH (%·h^−1/2^)	30.2	0.984
**EC**	kH (%·h^−1/2^)	30.51	0.986
**AG**	kH (%·h^−1/2^)	30.12	0.981
**AC**	kH (%·h^−1/2^)	30.27	0.988

## Data Availability

The original contributions presented in this study are included in the article. Further inquiries can be directed to the corresponding author.
